# Tensions in Remote Monitoring: Nurses’ Everyday Work in Digitally Supported Care

**DOI:** 10.1177/23333936261493300

**Published:** 2026-09-27

**Authors:** Melika Azim Zadegan, Päivi Eriksson, Eeva Aromaa, Tero Montonen

**Affiliations:** 1 University of Eastern Finland, Faculty of Social Sciences and Business Studies, Business School, Kuopio, Finland

**Keywords:** remote monitoring, nursing, work, tension, paradox, temporality, Finland

## Abstract

This study examines how nurses navigate home-based remote monitoring work in a hospital setting. Remote monitoring is increasingly used to support home-based care, yet how it reshapes healthcare professionals’ work is poorly understood. Drawing on qualitative interviews with nurses and doctors at a hospital clinic in Finland, an iterative process of interpretation and engagement with the data led to the development of five tensions between the present and future of remote monitoring work: (1) ongoing support versus anticipated patient autonomy, (2) added workload versus expected efficiency, (3) technical maintenance versus clinical interpretation, (4) selective eligibility versus broader inclusion, and (5) fragmented coordination versus integrated systems. Adopting a paradox perspective, the study treats these tensions as enduring rather than solvable. The findings show that the tensions are sustained through the relationship between present working conditions and anticipated developments in remote monitoring. The study conceptualises them as paradoxes constituted through communication, material arrangements, and the temporal connection between present work and anticipated futures.

## Introduction

Remote monitoring (RM) of patients in their homes is increasingly integrated into healthcare, reshaping how nurses work. Studies show that RM requires adjustments to routines, competencies, and responsibilities, as nurses engage in continuous data monitoring, patient communication, and coordination with other professionals (Anttila et al., 2023; Klemets et al., 2019; Van Rossum et al., 2021). In many settings, nurses are key users of RM systems, handling alerts, interpreting patient data, and communicating relevant information to physicians (e.g., Downey et al., 2018).

RM may support the early detection of changes in patients’ conditions, but it also introduces new ways of working, including ongoing observation, frequent follow-up, and the need for digital competence (Grøndahl et al., 2022; Van Grootven et al., 2023). RM also involves less visible nursing work, such as bringing together information from different sources and coordinating patients’ care trajectories (Allen, 2014). Digital systems can reshape professional roles and responsibilities, for example by transferring tasks between professional groups or increasing the responsibilities of staff with stronger digital competence (Lottonen et al., 2024).

Prior studies on nurses’ experiences of RM have mainly examined its implications for care delivery across different settings (Kawakami et al., 2025; Klemets et al., 2019; Kooij et al., 2022; Van Rossum et al., 2021). These studies document increased workload, coordination needs, and cognitive strain, but give less attention to how nurses navigate these demands in everyday RM work. Prior studies also refer to expectations of future improvements in RM, but these expectations are often treated as background assumptions but not examined as part of RM work (Rantz et al., 2015; Vaughan et al., 2024). Existing research therefore provides limited understanding of how nurses work with opposing demands as RM is introduced and developed in clinical practice.

In our study, we examine tensions in nurses’ RM work through a paradox perspective, which conceptualises opposing elements as interdependent and relatively persistent (Putnam et al., 2016). This perspective is useful for analysing tensions that cannot be resolved by prioritising one element over the other. We also attend to the technologies and organisational arrangements through which RM work is carried out (Barley, 1986, 2020). The study examines how nurses navigate RM work in a hospital setting. Doctors’ accounts are used to contextualise RM within broader clinical work, while the primary focus remains on nurses. The study contributes to research on paradoxes in nursing work by showing that tensions in RM are sustained through the connection between present working conditions and anticipated developments. It also shows how this connection is shaped by the material organisation of RM work.

## Theoretical framing

Research shows that nursing work is often characterised by tensions. Studies across different care settings describe instances of following protocols while exercising situational judgement, coordinating with others while taking individual responsibility, and maintaining professional distance while engaging emotionally with patients (Brik et al., 2026; Cohen et al., 2025; McGuire et al., 2006). These are part of daily practice because they are embedded in clinical decision-making and relational work and they tend to recur. Some studies conceptualise such tensions as paradoxes, highlighting that they persist over time and need to be navigated or coped with (Cohen et al., 2025). Research has also shown how such tensions shape how nurses understand their work, for example through oppositions such as care and control or standardisation and individualisation, as constructed in nurses’ accounts (McGuire et al., 2006).

However, less attention has been paid to the temporal aspects of tensions and to how they are shaped by the material conditions of digitally supported work. Digital technologies, protocols and organisational arrangements are often present in empirical descriptions, yet they are rarely examined as active elements in shaping tensions. Similarly, although tensions are recognised as ongoing, there is limited attention to how they are reproduced through work, and how their temporal dynamics extend beyond immediate situations, including how present conditions and challenges are related to anticipated future developments.

In many studies, the term ‘tension’ is used as a broad concept to capture dilemmas, trade-offs, and paradoxes (Putnam et al., 2016). Among these, paradoxes refer more specifically to relationships between opposing yet interdependent elements that persist over time and cannot be resolved definitively (Putnam et al., 2016). In our study, we draw on this distinction by conceptualising tensions as paradoxes. Putnam et al. (2016) approach paradoxes from a constitutive perspective, emphasising that paradoxes are not objective features of work life but are produced and sustained through communicative practices, such as how we describe, frame, and make sense of our work. At the same time, such communicative constructions are intertwined with organisational practices through which paradoxes are enacted and maintained. Therefore, we approach paradoxes as communicative and material. They emerge through how nurses make sense of their work, but also through the digital technologies and organisational arrangements.

We ground this in research showing that technologies are not neutral tools but part of how work is organised. In healthcare settings, technologies have been shown to shape roles, interactions and coordination practices (Barley, 1986). Barley (1986) demonstrates how the introduction of new technologies reconfigures patterns of interaction and authority, showing that material arrangements participate in structuring work. More recent work emphasises that technological change is closely intertwined with how work is carried out and experienced (Barley, 2020), highlighting how technologies become embedded in practices and contribute to the organisation of work. Tensions in nursing work are therefore both experienced through and shaped by material arrangements such as devices, software systems and care practices.

## Methods

Our study was guided by an interpretive research approach informed by reflexive orientation, acknowledging that findings are produced through an interpretive research process rather than discovered as objective facts (Braun & Clarke, 2019, 2021). Within this approach, we understand knowledge as contextually situated and co-produced through participants’ accounts and researchers’ interpretations of these accounts. This approach was appropriate because the study sought to understand how nurses attached meaning to their work with RM that was being piloted.

### Study context

We carried out the study at an outpatient clinic of a University Hospital in Finland, where a pilot project on home-based RM was underway for patients diagnosed with a chronic neurological condition that requires regular assessment and follow-up. During the pilot project, a wearable monitoring device was tested with a small group of older patients to assess its potential to support clinical evaluations between hospital visits and guide care decisions. Ethical approval for the study was granted by the Research Ethics Board of the North Savo Hospital District, Finland (Approval number: 276/2016 (5/2008)), with approval updated on June 22, 2021. Permission to conduct the study was granted by the participating University Hospital before data collection commenced. All healthcare professionals involved in the RM pilot were informed about the study and invited to participate voluntarily.

### Data collection

The study participants included two nurses responsible for the daily work with the RM pilot and one nurse with ten years of RM experience who served as a consultant in the pilot, five doctors whose patients were offered or might be offered home-based RM in the future, a medical resident and a physician trainee. A total of thirteen in-depth, thematic interviews lasting between 26–75 minutes were conducted between July 2022 and October 2023. Two nurses and the physician leading the pilot were interviewed twice, approximately eight months apart, to capture possible new insights as the pilot progressed. The remaining participants were interviewed once. The number of participants was appropriate for the analytical purpose of the study, which sought depth and contextual specificity in a single pilot setting. We considered the study to have sufficient information power (Malterud et al., 2016), as the research question was narrowly focused, participants were purposefully selected for their direct involvement in the RM pilot, and the interviews provided rich accounts of their work with RM. All participants received detailed information about the study and its publication and provided written informed consent.

Thematic interviews are organised around broad, open-ended topics that allow participants to elaborate on their experiences while ensuring that the same topics are addressed across interviews (Eriksson & Kovalainen, 2015). This approach was appropriate because the study followed an inductive, data-driven design. Instead of defining in advance which aspects of RM work were most important, the interviews gave participants the opportunity to emphasise the issues they considered most significant. The interview questions provided a common structure across interviews but also allowed the order and emphasis of the discussion to vary according to each participant’s experiences.

The interviews explored how RM was carried out in the pilot project, including practical tasks, coordination, and decision-making, as well as participants’ views on how RM work might develop in the future. Interviews were conducted either in Finnish or English by the three first authors of this article, and audio-recorded with participants’ permission. The interviews were carried out face-to-face at the hospital, with one conducted online. All interviews were transcribed verbatim, and those conducted in Finnish were translated into English by a professional translator. The accuracy of the translation was checked by the Finnish-speaking authors.

### Data analysis

The authors have backgrounds in qualitative organisational research and prior experience of collaborative research with healthcare professionals. None held an insider position in clinical work. Our backgrounds sensitised us to questions of work organisation, professional responsibilities, and competing demands, which may have influenced the development of themes formulated as tensions. We discussed developing interpretations in repeated analytic meetings and considered alternative readings of the data throughout the analysis (Braun & Clarke, 2021). We used reflexive thematic analysis (Braun & Clarke, 2019, 2021), treating theme development as an active process of interpretation. The initial analysis was inductive and grounded in participants’ accounts. It later became abductive as we developed and interpreted tensions through the lens of paradox theory. The analysis focused primarily on nurses because they carried out the day-to-day RM work described in the data. Doctors’ accounts provided an additional perspective, especially on anticipated developments, which were more prominent in their interviews.

In the first phase, we familiarised ourselves with all interviews by reading the transcripts several times and attending to how participants described RM as part of their everyday work. In the second phase, initial coding generated twenty semantic units that captured detailed meanings across the dataset. Coding was iterative and interpretive, as we moved back and forth between the data and our developing understanding of how professionals made sense of RM. The semantic units reflected participants’ accounts of RM-related work and their reflections on how this work might develop over time, including practical activities, coordination, technology use, and professional responsibilities. Consistent with reflexive thematic analysis, coding served as an analytic step through which we began to identify recurrent patterns of shared meaning across participants’ accounts.

In the third phase, we grouped the semantic units into ten broader categories that captured recurring patterns in how RM work was organised and experienced. This stage involved interpretive judgement as we considered how different coded segments related to each other and what kinds of opposing elements were embedded in participants’ accounts. We paid particular attention to how such opposing elements co-existed within the same situations, indicating recurring tensions in RM work. We examined how these oppositions formed enduring relations within participants’ accounts of everyday RM work. In this way, our analysis moved beyond topic summaries towards more interpretive thematic patterns, in a manner comparable to studies that have used reflexive thematic analysis to develop broader analytic structures from qualitative data (Jack et al., 2021).

In the fourth phase, we combined the broader categories into five main themes. Across the interviews, nurses described current challenges in RM work and referred to anticipated future developments. In the abductive phase, we drew on paradox theory to interpret empirically identified tensions as paradoxes, where one pole was embedded in present-day work practices and the other in an anticipated future. The abductive logic allowed us to analyse both empirical patterns and discuss their theoretical significance in detail (Table 1).

**Table 1.** Semantic Units, Categories, and Tensions in Remote Monitoring Work

Examples of semantic units	Broader category	Theme
Accounts of current support to patients in RM use	Ongoing support work enabling RM use	Ongoing support versus anticipated patient autonomy
Professional expectations of increasing patient independence	Anticipated shifts in patient capability and responsibility	
Accounts of current RM shaping workload, follow-up, and task distribution	RM as additional work	Added workload versus expected efficiency
Professional expectations of improved efficiency and workflow changes	Anticipated reorganisation of work through RM	
Accounts of current technology-related tasks as part of RM work	Technical maintenance as a precondition for RM	Technical maintenance versus clinical interpretation
Professional expectations of improved technological functioning and clinical interpretation	Anticipated shift towards clinically meaningful monitoring	
Accounts of current selection and assessment of patients for RM participation	Gatekeeping and selective participation	Selective eligibility versus broader inclusion
Professional expectations of broader participation enabled by system development	Anticipated expansion through system-level usability	
Accounts of current coordination, communication, and information flow in RM work	Organisational and informational fragmentation	Fragmented coordination versus integrated systems
Professional expectations of improved coordination and integration	Anticipated organisational and technical integration	

Source: Authors’ own work.

## Findings

The analysis resulted in five tensions in nurses’ accounts of RM work: (1) ongoing support versus anticipated patient autonomy, (2) added workload versus expected efficiency, (3) technical maintenance versus clinical interpretation, (4) selective eligibility versus broader inclusion, and (5) fragmented coordination versus integrated systems. Each tension is structured around how RM is currently experienced and how it is anticipated to develop, thereby sustaining the tension through anticipated futures. The descriptions of current RM work derive primarily from nurses’ accounts, whereas anticipated future developments are informed by both nurses’ and physicians’ perspectives. The following sections elaborate each tension in turn.

### Ongoing support versus anticipated patient autonomy

The first tension emerges in nurses’ accounts between the support currently required by patients monitored at home and the expectation of greater patient autonomy in the future. At present, nurses do not experience home-based RM as a self-managing practice but as support-intensive work that depends on sustained professional input, as Nurse 1 describes:

It takes a lot of support, the guidance and telephoning during the follow up period, going through things again and motivating them [patients] to use the equipment. They said a few times, ‘I think I won’t go on with this, it’s not working.’ So, I just say, ‘You are doing great, just a few days more.’ Encouraging them takes time.

Here, the nurse describes support as a normal feature of nursing work, indicating that RM is enabled through repeated contact, reassurance, and practical problem-solving with patients in their homes. Nurse 3 further outlines how nurses manage patient dependence by involving family members in device use and instruction: “When I instruct the patient on how to use the monitor, I instruct them both [patient and family caregiver] if they want me to”. At present, home-based RM is therefore experienced as dependent on continuous nursing support. However, nurses also articulated expectations of increased patient autonomy in the future, anticipating that the patient population will change, becoming more familiar with digital technologies and more capable of using RM with less professional input. Nurse 2 highlights this in the following way: “I do believe you shouldn’t underestimate old people when it comes to IT: [in the future] many of them will know how to use a smartphone and applications”.

Nurses do not describe the need for support as disappearing, but as potentially changing in how responsibility for RM use is distributed. These expectations are tentative and linked to broader changes in patient competence. The tension becomes visible in how present dependence and anticipated autonomy are described together without one replacing the other. Doctors also associated this tension with new forms of communication. As Doctor 4 explained, “I think in the future patients and caregivers can provide data there [RM application] and also communicate via this platform about ongoing health and technical issues, a new way of working”. This suggests that doctors expected greater patient autonomy through more active participation in communicating health information.

### Added workload versus expected efficiency

Nurses experienced RM as additional work during the pilot, although they expected it to improve efficiency if integrated into routine workflows. Nurse 2 describes how, at present, RM appears as additional work layered onto existing tasks: “It does add to my workload, because there is so much contact with the patient and the outpatient clinic appointments”. She also outlines expectations of future efficiency by linking these to how RM might become routinised and incorporated into clinical workflows: “I do not know if they can maybe skip some outpatient clinic visits. We might get that kind of benefit”.

Doctors associated this tension with the growing demands of clinical decision-making and professional learning. As the Medical Resident explained:

We have something new [RM data] to consider in clinical decision making, so it comes with a cognitive burden. In my role I also have to teach these things to my colleagues. This [RM] is happening on a small scale now, and if there is more of it, I will have to teach more and give more consultations. (Medical Resident)

For nurses in particular, expected efficiency remains located in a future where home-based monitoring has become part of routine workflows.

### Technical maintenance versus clinical interpretation

Technical maintenance occupied a substantial part of current RM work because devices, connections, and patient use had to be stabilised before monitoring data could be used clinically.

At present, Nurse 2 considers that home-based RM requires continuous attention to device functioning before monitoring data can be used clinically: “There is plenty to remember, you need to make sure the monitor and the phone do not run out of battery”. She describes how daily work is directed towards maintaining devices, connections, and patient use so that data can be generated at all. Technical troubleshooting is not occasional but ongoing, as Nurse 3 explains: “You cannot always be sure how the meter works… there have been lots of disconnections”. While emphasising how technical instability shapes what they are able to do clinically, nurses articulate expectations of a different future role for themselves, anticipating that improved technological reliability will allow them to focus more on clinical monitoring. Nurse 2 explains: “The idea is that when we will follow the data [in the future]… I will contact the patient if [his or her] movement begins to decrease”.

Doctors associated this tension with the clinical interpretation of RM data in the future. The Medical Resident explained: “If [patient] activity is getting lower, then we might have a problem with the shunt system, and that is clinically relevant because we have to address it”. This suggests that doctors anticipated RM becoming clinically valuable as monitoring data become sufficiently reliable to support clinical interpretation.

Technical instability therefore shapes what nurses and doctors are currently able to do clinically, while expectations of improved reliability locate meaningful data use in the future.

### Selective eligibility versus broader inclusion

Participation in RM was currently restricted to patients considered able and willing to use the technology, although participants expected improved usability and automation to enable broader inclusion. Nurse 2 describes present RM practice as a selective intervention that requires continuously assessing which patients are able and willing to use it: “There were certain conditions… you can’t find them [suitable patients] that easily”. The nurse describes how she acts as a gatekeeper who evaluates cognitive capacity, motivation, and practical ability of patients, making ongoing judgements about whether RM can be made to work for them. She highlights this as resource-intensive work, as inclusion requires effort to assess and support patients. Nurse 2 anticipated broader inclusion, expecting that improved usability would reduce the demands placed on individual patients: “Because [future device] will be easier to use, you could offer it to more people”.

Doctor 3 however associated this tension with future technological development: “In the ideal situation, the patient would not need to do anything. The monitoring would happen automatically in the background, and the information would come directly to us without requiring the patient to interact with the technology.” Thus, doctors anticipated broader participation in RM as monitoring technologies become simpler to use.

### Fragmented coordination versus integrated systems

Nurses described RM coordination as fragmented and their involvement in planning as limited, while both nurses and doctors anticipated more integrated information sharing in the future. Nurse 3, who has long experience with RM, notes how it is organised through partial information flows that depart from existing plans: “Well, there’s this fancy plan… who does what and when…”. In a similar way, Nurse 1 identifies nurses’ limited involvement in the RM pilot planning and decision-making: “We were not really involved… Everything is behind walls”.

At present, nurses experience that information about RM moves indirectly, which makes coordination reactive and leaves them without a shared overview of RM developments. However, Nurse 2 anticipated more integration and data sharing: “It is essential that information is communicated to the entire team”. Doctor 2 also associated future integration with better access to monitoring information as follows: “It could strengthen communication because everyone would have access to the same information instead of relying on separate contacts or fragmented reports”. Thus, both nurses and doctors anticipated RM becoming more integrated through shared access to patient information across healthcare professionals. Coordination remains reactive in the present, while integration is anticipated through future information sharing.

## Discussion

This study shows that tensions in RM work are sustained through the relationship between present working conditions and anticipated developments. We interpret these tensions as paradoxes constituted through nurses’ accounts, material arrangements, and the temporal orientation of RM work (Table 2). The following discussion examines how this interpretation extends research on paradoxes in nursing and digitally supported care.

**Table 2.** Temporal and Material Dimensions of Paradoxes in Nurses’ Work

Enacted paradox in RM work	Present work	Anticipated developments
Sustaining patients’ use of RM through ongoing support, while future autonomy is described as a possible development	The combination of RM technology and patients’ varying ability to use it requires continuous support and practical troubleshooting	Growing familiarity with smartphones, applications, and RM devices could reduce the need for continuous professional support
Managing additional RM work, while efficiency is anticipated as a possible outcome if RM becomes embedded in routine workflows	RM adds continuous follow-up activities to existing outpatient work	If integrated into routine workflows, RM could enable more targeted follow-up and reduce outpatient visits
Maintaining devices and data flows before clinical use of monitoring data is possible, while clinical work is described as dependent on more reliable technology	Maintaining reliable devices and uninterrupted data flows requires continuous work before monitoring data become clinically meaningful	More reliable technology could enable clinical interpretation of monitoring data
Ongoing assessment of which patients are able and willing to participate, while broader inclusion is described as dependent on improved usability	Current RM systems place practical and cognitive demands on patients, making participation selective	Improved usability and greater automation could enable wider patient participation
Working with partial information and unclear roles in coordinating RM, while more integrated coordination is a possible future development	RM information remains fragmented across systems, limiting access and leaving organisational responsibilities unclear	Integrated information systems and shared data access could enable more coordinated RM work

Source: Authors’ own work.

### From tensions to paradoxes in nursing work

Prior qualitative research has shown that nursing work is characterised by ongoing tensions, such as balancing protocols and professional judgement or care and control (Apker et al., 2005). Some studies have conceptualised these as paradoxes, emphasising their persistence (Cohen et al., 2025). Our findings extend this line of work by showing how such tensions in RM work are structured as enduring relations between opposing demands that are sustained in everyday work. Across the five identified tensions, opposing elements such as support and autonomy, workload and efficiency, technical maintenance and clinical interpretation, selective eligibility and broader inclusion, and fragmented coordination and integrated systems are experienced as interdependent elements of the same work. In this sense, the findings align with the definition of paradox as “contradictory yet interrelated elements that exist simultaneously and persist over time” (Putnam et al., 2016, p. 66). Moreover, these paradoxes are not resolved by eliminating one pole in favour of another but they are sustained through the combination of experiences and expectations. This supports prior research showing how nurses work with tensions through ongoing adjustment and sensemaking (Apker et al., 2005). The findings resonate with research showing that nurses’ work in hospital settings is not linear but cyclical, and organisationally entangled, requiring continuous practical adjustment in response to changing conditions (Hawkins & Morse, 2022). In this respect, home-based RM reconfigures nursing work that is already shaped by organisational practices.

### Temporal and material structuring of paradoxes

The findings contribute to understanding of paradoxes by showing how they are structured through temporal orientations and material conditions in nurses’ work. Prior research has recognised that tensions in nursing work are ongoing (Cohen et al., 2025), yet how they are sustained through the interplay of present material conditions and future-oriented expectations remains less explored. Our findings show that the paradoxes are temporally structured because present work is interpreted in relation to anticipated developments. Current support-intensive work is associated with possible future patient autonomy, and additional workload with expectations of efficiency once RM is integrated into routine workflows. These expectations do not remove current demands, but they provide a way of understanding them as part of an incomplete development process. In this sense, anticipated futures become interpretive resources through which present RM work is made manageable. This interpretation extends research on promissory digital health (Henwood & Marent, 2019; Marent & Henwood, 2022) by showing how anticipated digital futures shape present RM work.

These temporal orientations are intertwined with material conditions of work. As shown in Table 2, each tension is anchored in specific material arrangements in the present, such as devices, data flows, workflows, and information systems, and future-oriented expectations are linked to anticipated changes in these same conditions, including improved usability, system integration, and technological reliability. This highlights that paradoxes are temporally sustained and materially configured across present and anticipated futures of work.

A key contribution of this study is to show how paradoxes in nursing work are not only communicative but also material. Previous studies have largely focused on how tensions are articulated and constructed in interaction and discourse (McGuire et al., 2006). Our findings demonstrate how paradoxes are embedded in material arrangements, particularly in the digital technologies and other infrastructures of RM. For instance, the tension between technical maintenance and clinical interpretation is shaped by the functioning of devices, data flows, and system reliability. Nurses’ ability to enact clinical expertise then depends on stabilising technological conditions, which reconfigures what counts as clinical work. Similarly, tensions related to workload, inclusion, and coordination are shaped by how RM is organised through workflows, devices, software systems, and other infrastructures, although the role of materiality varies across tensions. In some cases, such as technical maintenance, material conditions are central to how the tension is experienced, whereas in others, such as support and autonomy, they form part of the background conditions that shape what is possible in practice. This finding resonates with Barley’s (1986) classic insight that technologies restructure work by redistributing tasks and redefining roles, as well as with later work showing how technological change is intertwined with everyday work (Barley, 2020).

### Implications for digitally supported nursing work

The findings also speak to broader research on nursing work. Remote monitoring, as documented here, draws on forms of coordination and interpretive work that have long been central to nursing practice, even when they remain invisible in formal accounts of care (Allen, 2014). In digitally mediated settings, nurses are continuously involved in sustaining continuity of care across shifting relations between patients, technologies, information, and organisational arrangements. RM extends these features of nursing work into new conditions without replacing them. This is consistent with research showing that nurses’ work is characterised by enduring tensions that require ongoing situated judgement and practical adjustment in everyday care (Apker et al., 2005; Cohen et al., 2025).

Finally, this study has limitations that open avenues for future research. The findings are based on a single pilot study, which limits transferability to more established forms of home monitoring. Future research could examine how paradoxes develop across longer implementation trajectories, in more established RM settings, or among professional groups whose work intersects with digital care. Also, the study did not examine the potential influence of sex or gender on participants’ experiences of RM work; future research could explore whether nurses’ and doctors’ experiences of digitally supported care vary in relation to sex and gender. From a practical perspective, the findings suggest that paradoxes in RM work are not temporary obstacles that can be resolved through technical or organisational fixes alone. Instead, they are ongoing features that need to be recognised and navigated in practice, which has implications for how digitally supported care interventions are designed, implemented, and supported.

## Conclusion

This study examined how nurses navigate home-based remote monitoring work in a hospital setting. It shows that this work is characterised by persistent tensions that connect present working conditions with anticipated developments in remote monitoring. These tensions are sustained through nurses’ accounts of their work and through the technologies and organisational arrangements on which that work depends. The study therefore extends understanding of paradoxes in nursing by showing how their temporal and material dimensions are intertwined in digitally supported care. Expectations of future improvement do not replace the demands of present work but become part of how these demands are understood and managed. Remote monitoring can thus be understood as an evolving form of work whose present arrangements remain closely connected to expectations of what it may become.

## Data Availability

The data supporting the findings of this study are not publicly available due to privacy restrictions that protect the confidentiality of the participants.
